# Advanced Emergency Braking Controller Design for Pedestrian Protection Oriented Automotive Collision Avoidance System

**DOI:** 10.1155/2014/218246

**Published:** 2014-07-06

**Authors:** Guo Lie, Ren Zejian, Ge Pingshu, Chang Jing

**Affiliations:** ^1^School of Automotive Engineering, Dalian University of Technology, Dalian 116024, China; ^2^College of Electromechanical & Information Engineering, Dalian Nationalities University, Dalian 116600, China

## Abstract

Automotive collision avoidance system, which aims to enhance the active safety of the vehicle, has become a hot research topic in recent years. However, most of the current systems ignore the active protection of pedestrian and other vulnerable groups in the transportation system. An advanced emergency braking control system is studied by taking into account the pedestrians and the vehicles. Three typical braking scenarios are defined and the safety situations are assessed by comparing the current distance between the host vehicle and the obstacle with the critical braking distance. To reflect the nonlinear time-varying characteristics and control effect of the longitudinal dynamics, the vehicle longitudinal dynamics model is established in CarSim. Then the braking controller with the structure of upper and lower layers is designed based on sliding mode control and the single neuron PID control when confronting deceleration or emergency braking conditions. Cosimulations utilizing CarSim and Simulink are finally carried out on a CarSim intelligent vehicle model to explore the effectiveness of the proposed controller. Results display that the designed controller has a good response in preventing colliding with the front vehicle or pedestrian.

## 1. Introduction

With the higher attention to the road traffic safety and the continuous development of the intelligent transportation systems worldwide, automotive active collision avoidance system, such as forward collision warning system, has become a hot research topic in recent years [1]. The automotive collision warning systems are able to warn the drivers actively when there is an imminent collision risk by providing the driver adequate time to take appropriate actions to reduce the severity of an accident. Some of these systems are equipped with autonomous braking, meaning that the vehicle will brake automatically if the driver does not respond in time. Then the effect of the collision can be mitigated or avoided. Preliminary studies suggest that such systems could ultimately save around 5,000 fatalities and 50,000 serious injuries per year across the EU [2]. However, most of the current automotive active collision avoidance systems take the leading vehicle as the collision avoidance targets and mainly focus on the safety of the host vehicles, ignoring the active protection of pedestrian and other vulnerable groups in the transportation system. Indeed, pedestrian protection is a key problem in the context of the automotive industry and its applications [3]. It is necessary to take into account a pedestrian as well as the leading vehicle or other kinds of obstacle when designing the collision avoidance system.

Fortunately, certain contributions have been made to enhance the active safety of pedestrian recently. For example, Fredriksson and Rosén [4] compared the potential pedestrian head injury reduction from current pedestrian passive and active safety countermeasures such as bonnet/airbag and autonomous braking. Llorca et al. [5] provided high precision GPS information for a driver, detected the pedestrian ahead by employing the stereo vision sensor, and designed steering controller for the pedestrian collision avoidance based on fuzzy logic. In view of vulnerable pedestrians on the road traffic accidents, Milanés et al. [3] designed a fuzzy control system with automatic steering focusing on pedestrian collision avoidance, realizing the braking behavior for pedestrian collision avoidance of intelligent vehicle. At the same time, they also pointed out that drivers are more likely to brake than to steer when confronting such emergency situations, although the optimal maneuver would be steering itself.

When designing the real-time control system, the vehicle dynamics model must be established necessarily for better reflection of the vehicle's actual running status. Considering the strong nonlinearity of the vehicle system and the uncertainty factors in the process of driving, the research on modeling and the design of the vehicle dynamic control has been conducted worldwide. For example, Ferrara and Vecchio [6], who were motivated by the robustness features of the sliding mode, designed a second-order sliding mode control system based on a nonlinear vehicle model. Zhu and Feng [7] presented a single neuron PID tracking control strategy for overtaking behavior. Nouvelière and Mammar [8] proposed an experimental implementation of a shared vehicle longitudinal controller based on a second-order sliding mode technique, which was tested under usual traffic conditions such as stop-and-go, stopping at obstacles, and car-following in a number of scenarios, but it mainly concentrated on the low velocity. Lee and Kim [9] controlled the relative velocity and distance of the vehicles by taking advantage of fuzzy logic theory. However, they ignored the influence of the dynamic characteristics of an engine model and the slip characteristics of the tire model, which is difficult to reflect the control effect in the real working conditions. Compared with fuzzy logic control, the sliding mode control and the neural network PID control have more strong robustness and can adapt to environmental changes. The fuzzy logic controller has to adjust a lot of parameters, and a good choice of rule base and parameters of membership functions is more important [10]. Majdoub et al. [11] built a nonlinear vehicle longitudinal motion model that accounted for most vehicle nonlinear dynamics including the tire-road interaction to ensure global stabilization and longitudinal velocity regulation during acceleration or deceleration driving modes.

Taking the forward pedestrians and vehicles as targets at the same time, an advanced emergency braking control system is presented for the vehicle collision avoiding system. The remainder of this paper is organized as follows.  Section 2 puts forward the braking control strategies and establishes the vehicle longitudinal dynamics model; the controller is designed when confronting deceleration or emergency braking conditions. Cosimulations utilizing CarSim and Simulink are finally carried out on a CarSim intelligent vehicle model in Section 3, which takes into account the longitudinal tire forces and tire dynamics, to explore the effectiveness of the proposed controller.  Section 4 concludes this paper.

## 2. Methodology

### 2.1. Braking Control Strategy

The automotive longitudinal active collision avoidance system includes the following key techniques: environment perception and processing, evaluation of the traffic safety state, vehicle dynamics modeling, and control execution techniques. The main purpose of environment perception and processing is to detect the vehicle running parameters in real time, using all kinds of sensors to obtain the accurate and reliable driving information after certain necessary signal processing.

After the detection of the pedestrians and vehicles ahead, the host vehicle must keep a safe distance from the front obstacle. Otherwise, it needs to be controlled when it is judged as danger. In this paper, the collision avoidance scenarios are simplified as follows [12]. Firstly, the dangerous obstacles ahead within the same lane are taken into account. Secondly, the autonomous brake maneuver is adopted instead of lane changing when a front vehicle or pedestrian emerges. Thirdly, the traffic safety is the main goal, ignoring the road traffic efficiency. Finally, the pedestrian velocity is negligible in reference to the velocity of the host vehicle and assumed to be zero in the same lane. Then the following three scenarios can be defined.

**Figure 1:**
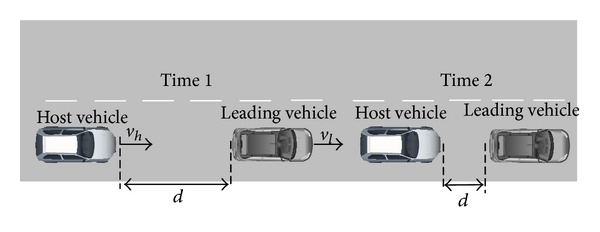
The leading vehicle decelerating and braking.

*(a) The Leading Vehicle Decelerating and Braking.* The traffic safety state is estimated on the basis of processing the leading vehicle information in the same lane; thus, a minimum safety distance model for collision avoiding is established based on the theoretical analysis on brake process of automobiles.  Figure 1 shows a schematic view of this scenario.

As shown in Figure 1, *d* represents the real-time distance between the host vehicle and the leading vehicle; if the host vehicle detects the leading vehicle decelerating, the host vehicle must decelerate or even brake to ensure a safety distance between each other. The critical braking distance can be obtained according to the safety distance model during the braking process as follows [13]:

(1) $$\begin{array}{c} d_{w1}=v_{h}t_{r}+\frac{\left(v_{h}-v_{l}\right)t_{i}}{2}+\frac{v_{h}^{2}-v_{l}^{2}}{2fg}+d_{\min}, \end{array}$$

where *d*_*w*1_ is the critical braking distance for decelerating, *v*_*h*_ and *v*_*l*_ represent the original velocity of the host and the leading vehicle, respectively, *t*_*r*_ is the sum of response time of the driver and braking coordination time ranging from 0.8 to 1.2 s, *t*_*i*_ is the growth time of the braking deceleration which varies from 0.1 to 0.2 s, *f* denotes the friction coefficient of the road, *g* is referred to as the acceleration of gravity, *d*_min⁡_ is the minimum distance between the host and the leading vehicle when they stop, ranging from 2 to 5 m.

After obtaining the critical braking distance, it is necessary to assess the current traffic safety state. According to (2), if the real-time distance *d* between the host vehicle and the leading vehicle is greater than the critical braking distance, the traffic state is safe and the vehicle can run with its current velocity [14]. Otherwise, if the driver does not decelerate or take other security measures when the current distance is lower than or equal to the critical braking distance, this state is judged to be dangerous and automatic braking deceleration on the host vehicle needs to be carried out immediately:

(2) $$\begin{array}{c} d>d_{w1},\;\text{safe}\\ d\leq d_{w1},\;\text{dangerous}. \end{array}$$

**Figure 2:**
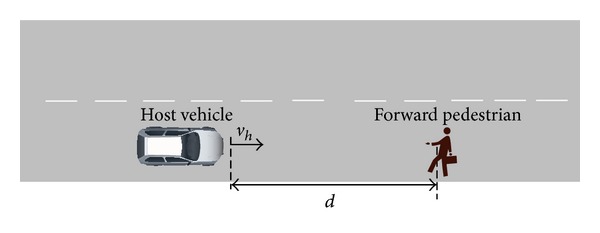
Pedestrian appears in front of the host vehicle.

*(b) Pedestrian or Static Obstacle Appears in the Same Lane.* Figure 2 shows a schematic view of a pedestrian appearing in front of the host vehicle. *d* represents the real-time distance between the host vehicle and the pedestrian ahead.

In the same way, the traffic safety state can be estimated according to the relative distance between the host vehicle and pedestrian ahead or obstacle in the same lane. The walking velocity of pedestrian is neglected in reference to the host vehicle and assumed to be zero. Then the critical braking distance between the host vehicle and the pedestrian could be calculated by

(3) $$\begin{array}{c} d_{w2}=v_{h}\left(t_{r}+\frac{t_{i}}{2}\right)+\frac{v_{h}^{2}}{2fg}+d_{\min}, \end{array}$$

where *d*_*w*2_ is the critical braking distance for decelerating and *d*_min⁡_ is the minimum distance between the host vehicle and the pedestrian when they stop, ranging from 2 to 5 m.

If the relative real-time distance *d* is larger than the critical braking distance, the vehicle could keep the original velocity and the pedestrian can cross the lane in safety. If the relative distance is lower than or equal to the critical braking distance, as shown in (4), and the driver still does not decelerate or take other security measures, this state is judged to be dangerous and automatic braking deceleration on the host vehicle needs to be implemented immediately:

(4) $$\begin{array}{c} d>d_{w2},\;\text{safe}\\ d\leq d_{w2},\;\text{dangerous}. \end{array}$$

*(c) Obstacles Appear Suddenly*. When the pedestrians from a very close distance are getting across the road quickly and fairly suddenly or the unexpected obstacles appear suddenly in front of the vehicle on the same lane when driving, we can obtain the real-time relative distance *d* which is too small between the obstacles and the host vehicle. This state, which meets the formula as follows, is judged to be extremely dangerous and needs to control the vehicle with the maximum braking deceleration in emergency circumstances at the same time:

(5) $$\begin{array}{c} d\leq \frac{d_{w2}}{2},\;\text{extremely dangerous.} \end{array}$$

### 2.2. Vehicle Model

*(a) The Vehicle Longitudinal Dynamics Model.* The vehicle dynamics simulation model is constructed on the basis of the CarSim software in this paper, including vehicle body, transmission system, braking system, suspension system, steering system, and tire system. Today, CarSim is one of the most widely used vehicle dynamics simulation packages in industry [15]. Adopting the CarSim vehicle model, one can simulate its real operation condition and reflect the system dynamic characteristic and balance model accuracy, making the simulation results better consistent with the real scene [16]. It is necessary to simulate the physical characteristics of the prototype parts accurately as much as possible in the modeling process in order to maintain the consistency of the characteristic of the CarSim model and the physical prototype.

The vehicle model can be built utilizing the CarSim software according to the structural parameters, as well as the model of the engine, transmission, steering, and other components. However, some parts cannot be defined in CarSim specifically, such as the characteristics of the brake system. For the reason that CarSim is able to interface with external MATLAB and Simulink easily, those parts can be defined in Simulink; therefore, the vehicle inverse longitudinal dynamics model should be built firstly.

*(b) The Vehicle Inverse Longitudinal Dynamics Model.* The desired throttle opening and brake pressure are calculated according to the requirements of the desired acceleration. They can be applied to vehicle dynamics system by the inverse engine model and braking actuator to control the acceleration and uniform motion of the vehicle [17]. Considering the situation that controlling the throttle and brake at the same time can lead to system shock in the actual driving process, it is needed to control the throttle and brake according to the desired acceleration [18].

We obtain the maximum acceleration *a*_max⁡_ under different velocity with CarSim by setting the value of throttle to be zero. In order to improve passenger's comfort and to prevent frequent switching between the throttle control and braking control during the controlling process, a buffer zone for the deceleration value is set [19]. Then the switching logic can be expressed as follows:

(6) $$\begin{array}{c} a_{\mathrm{des}}>a_{\max}+\Delta a,\;\text{throttle control}\\ a_{\mathrm{des}}\leq a_{\max}-\Delta a,\;\text{braking control,} \end{array}$$

where *a*_*des*⁡_ is the vehicle desired acceleration, *a*_max⁡_ is the maximum acceleration, and Δ*a* is the buffer value, which is set to 0.02 m/s^2^.

**Figure 3:**
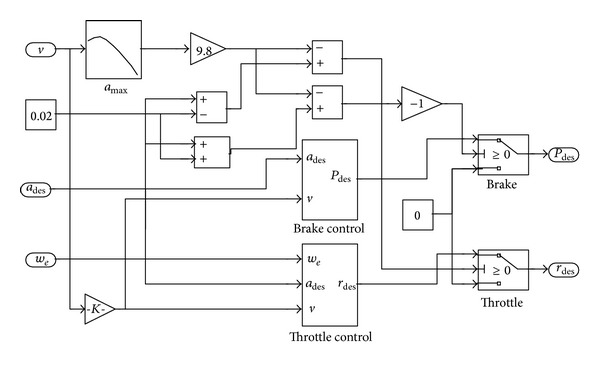
Switching logic schematic of the throttle control and brake control.

The switching schematic of throttle control and brake control which is built in Simulink is shown in Figure 3.

This switching logic schematic can choose the different control strategy by (6) according to the vehicle desired acceleration *a*_*des*⁡_ and export the desired braking pressure *P*_*des*⁡_ or the desired throttle opening *r*_*des*⁡_. Then the logic of subcomponents including the throttle control and the brake control is explained in detail as follows.

If the judging outcome is the throttle control, the desired engine torque can be calculated according to the requirement of the desired acceleration. Then the throttle aperture can be deduced from the inverse engine model. Ignoring the quality conversion of vehicles rotating parts, the vehicle longitudinal dynamics equation is as follows:

(7) $$\begin{array}{c} ma_{\mathrm{des}}=F_{t}-F_{xb}-\sum F\left(v\right), \end{array}$$

where *m* is the whole vehicle mass and *F*_*t*_ is the driving force of the vehicle from the road due to the driving effect of the engine, whose value is zero when braking. *F*_*xb*_ is the braking force on the vehicle from the road due to the effect of brake, whose value is zero when driving. ∑*F*(*v*) is the sum of resistance because of wind and rolling; namely,

(8) $$\begin{array}{c} \sum F\left(v\right)=\frac{1}{2}C_{D}A\rho v^{2}+mgf, \end{array}$$

where *ρ* denotes the air density, *C*_*D*_ represents the aerodynamic drag coefficient, *A* indicates the windward area, *v* is the vehicle velocity, and *f* denotes the friction coefficient of the road.

Ignoring the elastic deformation of the drive train, the vehicle driving force can be calculated by

(9) $$\begin{array}{c} F_{t}=\frac{\eta T_{e}\tau \left(w_{t}/w_{e}\right)R_{g}R_{m}}{r}, \end{array}$$

where *τ*(*w*_*t*_/*w*_*e*_) is the torque characteristic curve of the hydraulic torque converter, *T*_*e*_ is engine output torque, *R*_*g*_ is the transmission gear ratio, *R*_*m*_ means the main gearbox reduction ratio, *w*_*t*_ means the turbine velocity of the hydraulic torque converter, *w*_*e*_ is the engine velocity, *η* denotes the mechanical efficiency, and *r* is the tire rolling radius.

*K*_*d*_ is a variable and is defined as

(10) $$\begin{array}{c} K_{d}=\frac{\eta \tau \left(w_{t}/w_{e}\right)R_{g}R_{m}}{r}. \end{array}$$

The braking force *F*_*xb*_ is zero when driving; then, by formulas (7), (8), (9), and (10), the desired engine output torque is obtained as follows:

(11) $$\begin{array}{c} T_{e}=\frac{F_{t}}{K_{d}}=\frac{ma_{\mathrm{des}}+\left(1/2\right)C_{D}A\rho v^{2}+mgf}{K_{d}}. \end{array}$$

When acquiring the desired engine output torque *T*_*e*_, the engine velocity *w*_*e*_, and the reverse engine torque characteristic curve *f*(*T*_*e*_, *w*_*e*_), the desired throttle opening *r*_*des*⁡_ can be obtained accurately:

(12) $$\begin{array}{c} r_{\mathrm{des}}=f\left(T_{e},w_{e}\right). \end{array}$$

If the judgment result is the brake control, the desired braking torque can be calculated according to the requirements of the desired acceleration; then transform the desired braking torque into the brake pressure based on a linear relationship between braking torque and the brake pressure.

Under the maximum braking force of the road, the braking force can be approximately expressed as a linear function of the oil pressure in the braking line, and its expression is as follows:

(13) $$\begin{array}{c} \frac{T_{\text{bf}}+T_{\text{br}}}{rP}=\frac{F_{b}}{P}=K_{b}, \end{array}$$

where *T*_bf_ and *T*_br_ represent the braking torque of the front and rear wheels, respectively, *r* is the tire rolling radius, *F*_*b*_ expresses the braking force, and *K*_*b*_ is the ratio of the brake force and brake pressure.

The driving force *F*_*t*_ is zero when braking, so the desired brake pressure *P*_*des*⁡_ can be calculated as

(14) $$\begin{array}{c} P_{\mathrm{des}}=\frac{\left|ma_{\mathrm{des}}+\left(1/2\right)C_{D}A\rho v^{2}+mgf\right|}{K_{b}}. \end{array}$$

### 2.3. Controller Design

The design of control system is the key of the automatic stopping research, which aims to realize its function by the control of the vehicle longitudinal dynamics [20]. Two controllers are designed in this paper, the braking controller for deceleration and the braking controller for emergency, with the aim of automatic stopping control under different scenarios.

**Figure 4:**
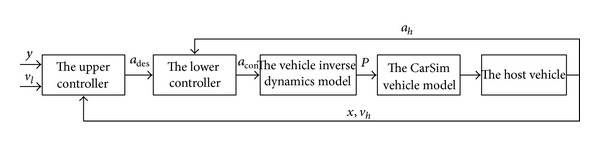
Scheme of the braking controller for deceleration.

*(a) Design of the Braking Controller for Deceleration.* The braking controller for deceleration employs a hierarchical control structure. The upper controller determines the expectations of the acceleration of the host vehicle in the current situation according to the requirement that proper distance should be kept between the leading vehicle and the host one in the same lane. The lower controller controls the vehicle dynamics system to achieve the desired acceleration on the basis of the output of the upper controller. The vehicle dynamics system which was introduced in Section 2.2 includes the inverse dynamics model and the vehicle model with CarSim. The scheme of the braking controller for deceleration is shown in Figure 4 and the detailed design process of the controller, which includes the upper one and the lower one, is introduced below.

In the upper controller, we obtain the expectations of the acceleration by adopting the method of the sliding mode control. Besides, the sliding mode control methodology has the advantage of producing low complexity control laws, which appear particularly suitable to be implemented in the electronic control unit (ECU) of the controlled vehicles. Sliding mode control methodology [21, 22] has its capacity to completely reject the effect of bounded uncertainty acting in the input channels, the so-called matched uncertainty [23]. So when designing the upper controller of desired acceleration, the sliding mode control methodology is adopted to solve the problems of the system's dynamics caused by the uncertainties of various natures in the automotive context. In order to enhance the robustness against unmatched perturbations, endeavors can be done by combination of sliding mode technique with other robust strategies, such as *H*_*∞*_ and backstepping [24]. Moreover, the controller in operation can compensate a number of physical effects which are neglected by the simple model of the vehicle to make the design of the controller feasible. The same holds for disturbances of different types, as well as for parameter variations.

Relative distance error is an important index of the control system evaluation. In order to improve the control precision of the model, this paper targets the relative distance and relative velocity error between the obstacles ahead and the host vehicle as the two indices of control system [8]. Define variable parameters of the upper controller as follows:

(15) $$\begin{array}{c} \varepsilon =y-x-\left(l_{0}+v_{l}t_{0}\right),\\ \dot{\varepsilon }=v_{l}-v_{h}, \end{array}$$

where *ε* and $\dot{\varepsilon }$ signify the errors of the relative distance and the relative velocity, respectively; *y* and *x* signify the longitudinal position of the target obstacle and the host vehicle, respectively; *v*_*l*_ and *v*_*h*_ denote the longitudinal velocity of the target obstacle and the host vehicle, respectively; *l*_0_ is the distance between the obstacle and the host vehicle after the successful collision avoidance; *l*_0_ is set to 6 m; *t*_0_ is the headway time, whose value is 1.5 s.

According to the theory of sliding mode control [25, 26], the switching surface based on trending law in a limited time for the collision avoidance control system is defined as follows [27]:

(16) $$\begin{array}{c} S\left(t\right)=\dot{\varepsilon }-\lambda_{1}\varepsilon -\lambda_{2}\overset{t}{\underset{0}{\int }}\varepsilon \;dt, \end{array}$$

where *λ*_1_ > 0 and *λ*_1_ > 0 are two parameters of sliding mode control. The derivative of (16) is

(17) $$\begin{array}{c} \dot{S}\left(t\right)={\dot{v}}_{l}-{\dot{v}}_{h}-\lambda_{1}\dot{\varepsilon }-\lambda_{2}\varepsilon . \end{array}$$

The influence of the friction, external disturbance, and parameter perturbation are difficult to avoid in the actual system; thus, choosing an appropriate control law, adopting the symbol function sgn (*S*), needs to be considered at this time in order to make the first derivative of sliding mode switching surface *S* convergent; namely,

(18) $$\begin{array}{c} \dot{S}=-\beta \mathrm{sgn}\left(S\right),\;\beta >0. \end{array}$$

Then the desired acceleration of the host vehicle can be obtained as

(19) $$\begin{array}{c} a_{\mathrm{des}}={\dot{v}}_{h}={\dot{v}}_{l}+\lambda_{1}\dot{\varepsilon }+\lambda_{2}\varepsilon -\beta \mathrm{sgn}\left(S\right). \end{array}$$

To analyze the stability of the controller, a Lyapunov function is defined as follows:

(20) $$\begin{array}{c} V_{2}=\frac{1}{2}S^{2}. \end{array}$$

Obviously, *V*_2_ > 0, differentiating *V*_2_ as

(21) $$\begin{array}{c} {\dot{V}}_{2}=S\dot{S}=-S\cdot \beta \mathrm{sgn}\left(S\right)=-\left|S\right|\cdot \beta . \end{array}$$

According to the Lyapunov stability criterion, when *β* > 0, apparently ${\dot{V}}_{2}<0$ for all *t* ∈ (0, +*∞*), the control system is stable and can effectively restrain and weaken the buffeting of the system, which has a good robustness against the interference outside.

In the lower controller, the desired acceleration *a*_*des*⁡_ is obtained by (19) and then takes the difference between the value of the desired acceleration *a*_*des*⁡_ and the current actual acceleration *a*_*h*_ as the input of the single neuron PID controller.

A single neuron adaptive intelligent PID controller consisting of the single neuron with the self-learning and adaptive capacity not only has simple structure, but also can adapt to environmental changes and has strong robustness. The driver controls the longitudinal acceleration of the vehicle by controlling the position of the brake pedal and then controls vehicle longitudinal velocity in the process of the actual driving. This control system becomes a strongly nonlinear parameter time-varying system due to involvement of the strongly nonlinear link of the brake and tire. Thus, a single neuron PID controller, which is suitable for the nonlinear control, is applied to the lower controller of the collision avoidance control system, to meet the requirements of the system's precision and response.

The connection weights of the controller are adjusted directly utilizing the improved supervised Hebb learning rule in the literature [28], so as to achieve the online self-tuning of the single neuron PID controller parameters, ensuring the adaptability and robustness of the controller.

The state variables of *x*_1_, *x*_2_, and *x*_3_ required in the single neuron controller are as follows [7]:

(22) $$\begin{array}{c} x_{1}\left(t\right)=e\left(t\right)=a_{\mathrm{des}}\left(t\right)-a_{h}\left(t\right)\\ x_{2}\left(t\right)=\Delta e\left(t\right)=e\left(t\right)-e\left(t-1\right)\\ x_{3}\left(t\right)=e\left(t\right)-2e\left(t-1\right)+e\left(t-2\right)=e\left(t-1\right)-e\left(t-2\right). \end{array}$$

Control algorithms and learning rules are shown in the following equation:

(23) $$\begin{array}{c} a_{\text{con}}\left(t\right)=a_{\text{con}}\left(t-1\right)+K\overset{3}{\underset{i=1}{\sum }}w_{i}^{\prime }\left(t\right)x_{i}\left(t\right)\\ w_{i}^{\prime }\left(t\right)=\frac{w_{i}\left(t\right)}{\sum_{i=1}^{3}\left|w_{i}\left(t\right)\right|}\\ w_{1}\left(t\right)=w_{1}\left(t-1\right)+\mu_{I}e\left(t\right)a_{\text{con}}\left(t-1\right)\left(e\left(t\right)+\Delta e\left(t\right)\right)\\ w_{2}\left(t\right)=w_{2}\left(t-1\right)+\mu_{P}e\left(t\right)a_{\text{con}}\left(t-1\right)\left(e\left(t\right)+\Delta e\left(t\right)\right)\\ w_{3}\left(t\right)=w_{3}\left(t-1\right)+\mu_{D}e\left(t\right)a_{\text{con}}\left(t-1\right)\left(e\left(t\right)+\Delta e\left(t\right)\right), \end{array}$$

where *μ*_*I*_, *μ*_*P*_, and *μ*_*D*_ signify the learning rates of integral, proportional, and differential components, respectively. *K* denotes the proportion coefficient of neurons such that *K* > 0, *w*_*i*_(*t*) corresponds to the weighting coefficient of *x*_*i*_(*t*), *a*_*des*⁡_(*t*) is the desired acceleration, *a*_*h*_(*t*) is the actual acceleration of the host vehicle, and *a*_con_(*t*) is the controlled acceleration.

In this paper, the learning rates *μ*_*I*_, *μ*_*P*_, and *μ*_*D*_ are set to different value in order to adjust different weight coefficients singly. The online correction of the weighting coefficient is not entirely based on neural network learning algorithm but refers to the actual situation, better meeting the requirements of real-time and accuracy.

**Figure 5:**
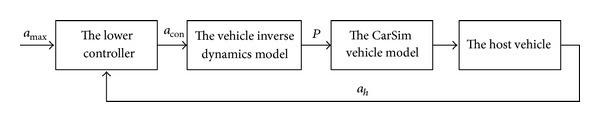
Scheme of the braking controller for emergency.

*(b) Design of the Braking Controller for Emergency.* When the vehicle is in the state of extreme danger, the braking controller for deceleration may fail to realize collision avoidance. Therefore, an emergency braking controller is necessary to ensure the safety of vehicle and pedestrian. Emergency braking needs to be carried out in an extremely dangerous state with the maximum braking deceleration to avoid a collision. A single neuron PID controller similar to the controller for braking deceleration is designed. It takes the maximum value of the braking deceleration as the desired braking deceleration to control vehicle dynamical system so as to achieve the desired braking deceleration and stop the vehicle achieving certain safety distance from the obstacle. The maximum braking deceleration of the vehicle is −8.5 m/s^2^. The scheme of a braking controller for emergency is shown in Figure 5.

## 3. Simulation Results

To test and verify the effect of the controller, three typical scenarios are presented for cosimulation utilizing CarSim and Matlab/Simulink. The simulation time step and frequency are 0.001 s and 1000 Hz, respectively. This paper chooses the main parameters of the intelligent vehicle prototype of Dalian University of Technology (DUTIV) developed in our research group to build models using CarSim. The parameters of the DUTIV intelligent vehicle prototype are shown in Table 1. The parameters of the CarSim vehicle model are set according to Table 1. The longitudinal velocity, acceleration, and location information of the vehicle model are transmitted from CarSim to Simulink.

The parameters of the designed controller for deceleration are shown in Table 2.

The parameters of the designed controller for emergency are shown in Table 3.

### 3.1. Deceleration Braking Control for Vehicle Collision Avoidance

**Figure 6:**
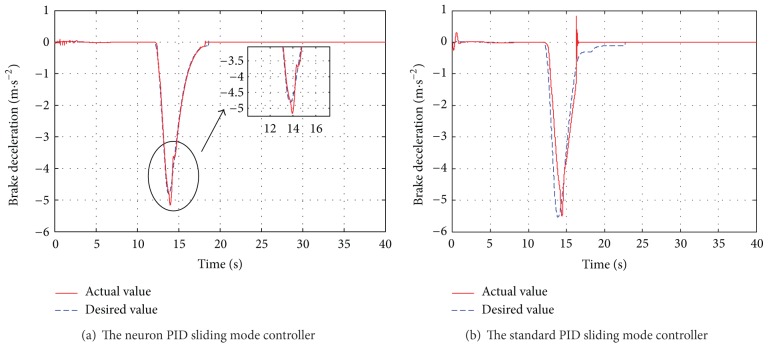
The vehicle deceleration.

**Figure 7:**
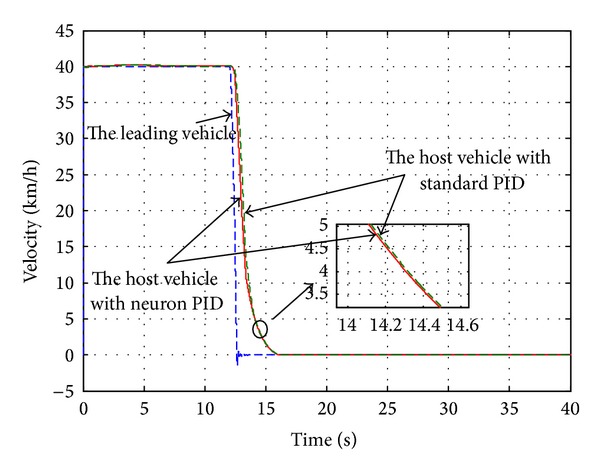
The vehicle velocity.

**Figure 8:**
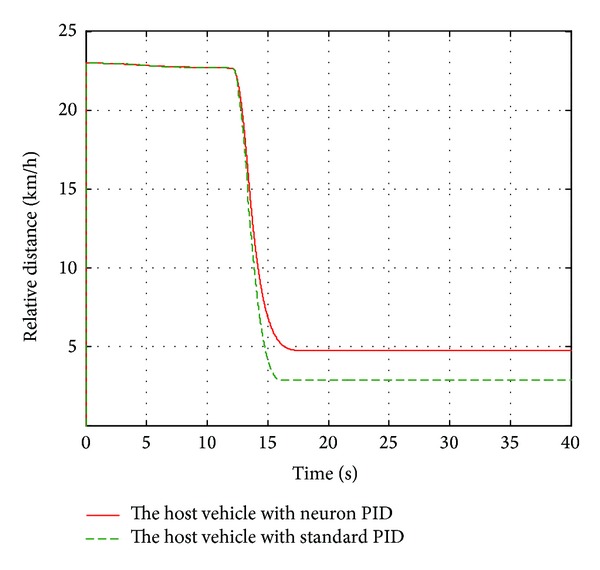
The relative distance.

In this situation, the host vehicle is running at a constant velocity of 40 km/h. The leading vehicle is running at a constant velocity of 40 km/h and conducts the maximum intensity of emergency braking during the time range of 12 s and 12.3 s. The leading vehicle is detected when it is 23 m ahead of the host vehicle. When the real-time distance *d* between the host vehicle and the leading vehicle appears to be smaller than the critical braking distance *d*_*w*1_, which is introduced in Section 2.1(a), the automatic deceleration control starts to be implemented immediately. During the simulation, the initial position of the leading vehicle is set to 23 m away from original point and the initial velocity is set to 40 km/h and the brake pressure steps from 0 Mpa to 15 Mpa. The host vehicle starts to move from the original point with a constant velocity of 40 km/h. The performance of the designed neuron PID sliding model controller is compared with the standard PID sliding mode controller. The simulation duration is set to 40 s to observe the changes of velocity, acceleration, and relative distance. The simulation results are shown in Figures 6–8.

Figure 6 represents the simulation results using two different controllers.  Figure 6(a) shows that the actual braking deceleration is nearly accordant with the desired braking deceleration using the single neuron PID based sliding mode controller. In order to avoid collision, the host vehicle braking deceleration increases gradually following the desired one at 12.1 s and reaches the maximum braking deceleration of −5.1 m/s^2^.  Figure 6(b) shows the results of the braking deceleration based on standard PID sliding mode controller. Seen from Figure 6(b), the actual braking deceleration has a certain degree of lag to the desired value between 12.5 s and 14.5 s. The difference between the actual braking deceleration and the desired braking deceleration varies widely from 16 s to 23 s.

As shown in Figure 7, the velocity of the host vehicle based on the single neuron PID sliding mode controller declines rapidly when the leading vehicle brakes and slows down to zero at 18 s, while the leading vehicle slows down to zero at 16 s. The velocity of the host vehicle based on standard PID sliding mode controller is slower than that of the single neuron PID sliding mode controller. From Figure 8, it can be known that the relative distance remains unchanged approximately before the leading vehicle brakes absolutely. It decreases continuously when the leading vehicle decelerates. Finally the distance between two vehicles based on single neuron PID sliding mode controller remains 4.78 m until they stop completely, meeting the requirement of the safe distance and ensuring the traffic safety, while this distance based on standard PID sliding mode controller remains 2.88 m, which is in a dangerous situation.

### 3.2. Deceleration Braking Control for Pedestrian Collision Avoidance

**Figure 9:**
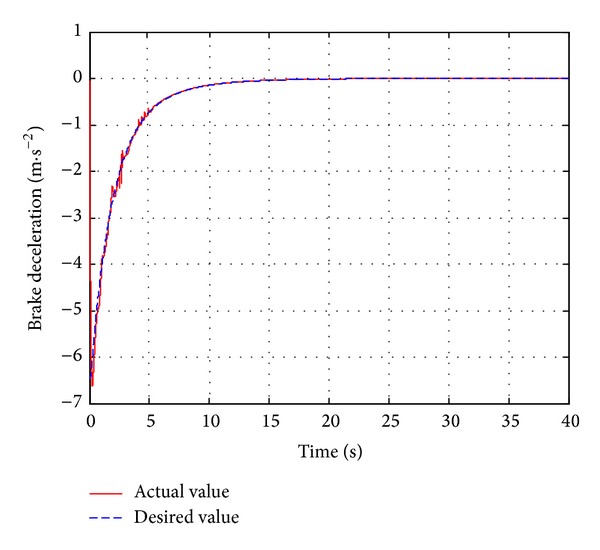
The vehicle deceleration.

**Figure 10:**
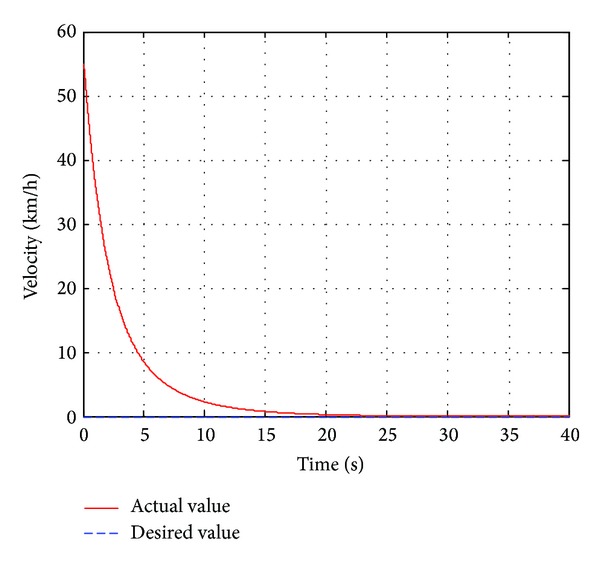
The vehicle velocity.

**Figure 11:**
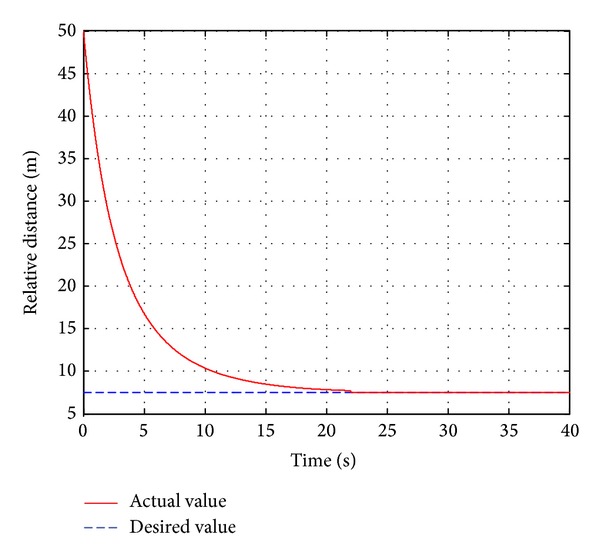
The relative distance.

In this case, the pedestrian's walking velocity is neglected in reference to the host vehicle and assumed to be zero. The pedestrian is detected 50 m ahead of the host vehicle, which is running at a constant velocity of 55 km/h. When the real-time distance *d* between the host vehicle and the detected pedestrian appears to be smaller than the critical distance *d*_*w*2_, which is introduced in Section 2.1(b), the automatic deceleration control starts to be implemented immediately. Before the simulation, the initial position of the pedestrian is placed 50 m ahead of the original point. The host vehicle starts to move from the original point with a constant velocity of 55 km/h. The simulation duration is set to 40 s in CarSim. The simulation results are shown in Figures 9–11.

As shown in Figure 9, the actual braking deceleration is nearly accordant with the desired braking deceleration, and the actual braking deceleration reaches the maximum braking deceleration −6.6 m/s^2^ at 0.25 s. At that time, the passengers would feel uncomfortable to some extent. Some little fluctuations can be observed between 2 s and 5 s. The actual braking deceleration reaches zero at 22 s. From Figure 10, it can be known that the velocity drops rapidly within the first five seconds and reduces to 9 km/h at the time of 5 s. At the time of 15 s the velocity is already reduced to 1.2 km/h and finally reaches the desired value of zero at 22 s.  Figure 11 shows that the relative distance of the vehicle and the target pedestrian is 50 m at the initial time and quickly reduces to 16.5 m within the first five seconds. Finally, the relative distance reaches the desired distance at the time of 22 s when the host vehicle stops completely.

### 3.3. Emergency Braking Control for Pedestrian Collision Avoidance

**Figure 12:**
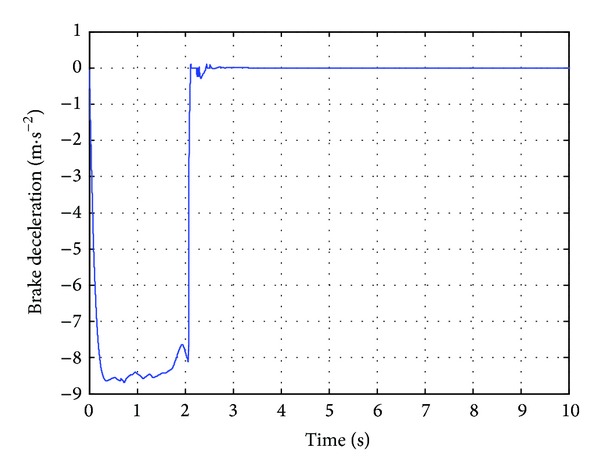
The vehicle deceleration.

**Figure 13:**
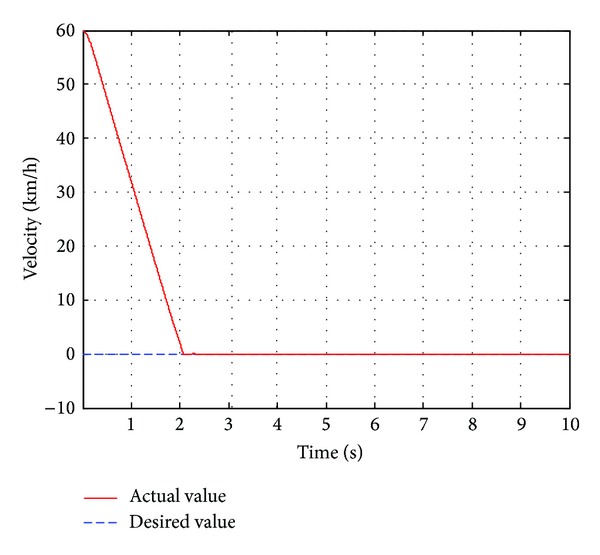
The vehicle velocity.

**Figure 14:**
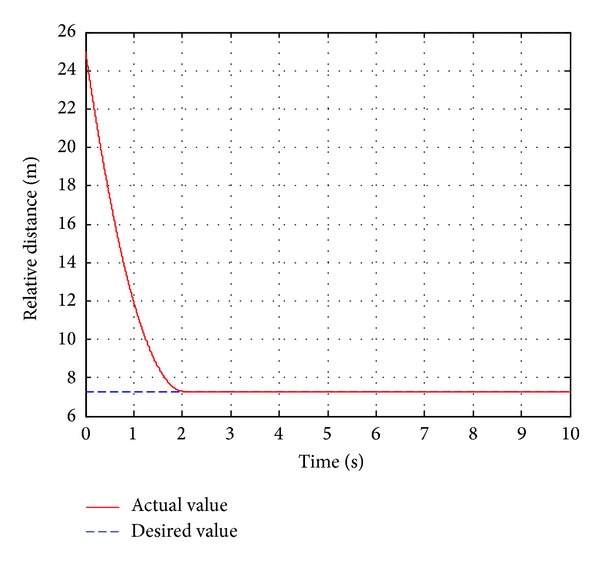
The relative distance.

In this case, the pedestrian's walking velocity is neglected in reference to the host vehicle and assumed to be zero. The pedestrian is detected 25 m ahead of the host vehicle, which is running at a constant velocity of 60 km/h. The pedestrian is in extremely dangerous state after the traffic safety state estimation, and the host vehicle should be controlled for emergency collision avoidance. Before the simulation, the initial position of the pedestrian is placed 25 m ahead of the original point. The host vehicle starts to move from the original point with a constant velocity of 60 km/h. The simulation duration is set to10 s in CarSim. The simulation results are shown in Figures 12 to 14.

As shown in Figure 12, the actual braking deceleration reaches the maximum braking deceleration of −8.5 m/s^2^ immediately at 0.3 s. Some little fluctuations can be observed between 0.3 s and 2 s. At that time, the passengers could feel uncomfortable to some extent. Then the actual braking deceleration drops rapidly and quickly reaches zero.  Figure 13 shows that the velocity drops rapidly with maximum braking deceleration, from the initial velocity of 60 km/h to 2 km/h at 2 s, and reaches zero at 2.3 s when the vehicle stops. From Figure 14, we could know that the relative distance of the vehicle and the pedestrian was 25 m at the initial time and gradually tends to the desired relative distance. Finally, the relative distance reached 7.2 m when the host vehicle stops completely.

## 4. Conclusion

According to the requirement of the collision avoidance control system, the safety status was judged by comparing the current distance between the host vehicle and the obstacle with the critical braking distance. Then the vehicle dynamics model and the vehicle inverse longitudinal dynamics model were established in CarSim. The collision avoidance controller was designed based on sliding mode and single neuron PID method, realizing automatic braking control of the host vehicle for vehicle or pedestrian collision avoidance and guaranteeing the active safety of the vehicle. The parameters of the experimental vehicle were applied to the vehicle model utilizing CarSim, which could better reflect nonlinear time-varying characteristics and control effect of the longitudinal dynamics, possessing a certain practical significance and research value. Finally, cosimulations were carried out on a CarSim vehicle model utilizing Matlab/Simulink to explore the effectiveness of the proposed controller. Results indicate that the controller can realize the deceleration or emergency brake when there is a pedestrian or vehicle ahead of the host vehicle. However, more possible collision avoidance scenarios should be taken into account. Additionally, the proposed controller should be applied to the real intelligent vehicle prototype which also can deal with the emergency situation of more collision avoiding scenarios and consider other measures like braking and lane changing at the same time in the future work.

## Figures and Tables

**Table 1:** DUTIV intelligent vehicle parameters.

Symbol	Designation	Value
*f*	rolling friction coefficient	0.014
*r*	wheel rolling radius	0.3495/m
*I*_*w*_	wheel rotational inertia	1.6/kg*·*m^2^
*I*_*z*_	moment of inertia around the *z* axis	3059/kg*·*m^2^
*m*	whole vehicle mass	1390/kg
*C*_*D*_	aerodynamic drag coefficient	0.32
*a*	distance from front axle to vehicle mass center	1.463/m
*b*	distance from the rear axle to the mass center	1.047/m
*H*_*g*_	height of the mass center of the vehicle	0.58/m
*B*_*f*_	front wheel base	1.524/m
*B*_*r*_	rear wheel base	1.524/m
*A*	windward area	2.674/m^2^

**Table 2:** Control parameters for deceleration brake.

Symbol	*λ*_1_	*λ*_2_	*β*	*μ*_*I*_	*μ*_*P*_	*μ*_*D*_	*K*
Value	0.69	0.1	0.012	20	500	500	0.3

**Table 3:** Control parameters for emergency brake.

Symbol	*μ*_*I*_	*μ*_*P*_	*μ*_*D*_	*K*
Value	20	500	500	0.3
